# Identification of Sphingosine Kinase 1 as a Novel Protein Regulated by High Molecular Weight Hyaluronan in Ovarian Cancer

**DOI:** 10.1111/jcmm.70574

**Published:** 2025-05-12

**Authors:** Zoe K. Price, Noor A. Lokman, Jessica Morrison, Sisanda N. Mhlanga, Mai Sugiyama, Yoshihiro Koya, Lorena T. Davies, Stuart M. Pitson, Martin K. Oehler, Melissa R. Pitman, Masato Yoshihara, Hiroaki Kajiyama, Carmela Ricciardelli

**Affiliations:** ^1^ Discipline of Obstetrics and Gynaecology Adelaide Medical School, University of Adelaide Adelaide South Australia Australia; ^2^ Robinson Research Institute University of Adelaide Adelaide South Australia Australia; ^3^ Department of Obstetrics and Gynecology Nagoya University Graduate School of Medicine Nagoya Japan; ^4^ Centre for Cancer Biology University of South Australia and SA Pathology Adelaide South Australia Australia; ^5^ Molecular and Biomedical Science, Molecular Life Sciences University of Adelaide Adelaide South Australia Australia; ^6^ Department of Gynaecological Oncology Royal Adelaide Hospital Adelaide South Australia Australia

**Keywords:** 4‐methylumbelliferone, hyaluronan, Notch3, ovarian cancer, SPHK1

## Abstract

The effects of hyaluronan (HA) in cancer are widely studied; however, the role of different molecular weight HA is poorly understood. Identifying novel proteins regulated by different molecular weight HA may highlight novel therapeutic targets. Proteomics analysis was performed to identify novel proteins regulated by different molecular weight HA (27, 183 and 1000 kDa) in ES‐2 ovarian cancer cells over‐expressing Notch3 intra‐cellular domain. Our analyses identified sphingosine kinase 1 (SPHK1), a novel protein regulated by 183‐ and 1000‐kDa HA. Utilising online databases and high‐grade serous ovarian cancer (HGSOC) patient tissue microarray cohorts, we assessed the relationship between SPHK1 expression and ovarian cancer metastasis, recurrence and patient outcome. We assessed the effects of the HA synthesis inhibitor 4‐methylumbelliferone (4‐MU) on SPHK1 expression in ovarian cancer cells and HGSOC patient tissues using ex vivo tissue explant assays. SPHK1 was significantly increased in ovarian cancer compared to normal tissues, elevated in metastatic and recurrent HGSOC tissues and associated with poor patient outcome. 4‐MU significantly inhibited SPHK1 expression in ovarian cancer cells (ES‐2, CaOV3 and A2780) and HGSOC patient tissues. This study highlights a link between HA and SPHK1 expression in ovarian cancer. Our findings confirm an adverse effect on ovarian cancer prognosis. SPHK1 constitutes a novel promising target against ovarian cancer that warrants further investigation.

**Abbreviations**
4‐MU: 4‐methylumbelliferone
EMT: epithelial to mesenchymal transition
FT: fallopian tube
HA: hyaluronan
HMW‐HA: high molecular weight HA
NICD3: Notch3 intra‐cellular domain
SPHK1: sphingosine kinase 1

## 1 Introduction

Ovarian cancer is the most lethal gynaecological cancer in countries with a very high development index [1]. 90% of ovarian cancers are of epithelial origin, with 60%–70% of malignant epithelial ovarian tumours belonging to the high‐grade serous ovarian cancer (HGSOC) sub‐type [2]. Primary treatment for ovarian cancer consists of debulking surgery followed by combination chemotherapy. 85% of HGSOC patients respond to initial treatment, but unfortunately 75% experience recurrence and develop acquired resistance to cytotoxic therapies [2]. New treatment strategies are required to overcome chemotherapy resistance.

HA, a glycosaminoglycan, is frequently upregulated in cancer. It is shown to elicit a range of pro‐tumourigenic signals including enhanced cell proliferation, migration and therapy resistance in ovarian cancer [3]. We have previously demonstrated serum HA levels are significantly enhanced in patients with chemotherapy resistant compared to chemosensitive disease [4]. Biologically, HA is present as a wide range of different molecular weight polymers, from single disaccharides to polymers over 3000 kDa. It is well known that low molecular weight HA (LMW‐HA) is pro‐inflammatory and pro‐angiogenic, whereas high molecular weight HA (HMW‐HA) is anti‐inflammatory and anti‐angiogenic [3]. A study by Shiina et al. showed that 200‐kDa HA enhanced spheroid formation and expression of stem cell markers in a population of head and neck cancer cells (HSC‐3) selected for cancer stem cell markers ALDH and CD44v3 [5]. We recently investigated the effects of different molecular weight HA in an ovarian cancer stem cell model by over‐expressing Notch3 intra‐cellular domain (NICD3) in ES‐2 cells [6]. Notch3 stimulates stem‐associated features and increases side‐population cells, which are enriched for cancer stem cell markers [7]. Interestingly, we found this population of cells was more responsive to 1000‐kDa HA when combined with ES‐2 wild‐type (WT) cells (ES‐2:ES‐2‐Rv‐NICD3, 1:3) as described in our recent study [6]. 1000‐kDa HA significantly increased spheroid formation by ES‐2:ES‐2‐Rv‐NICD3 (1:3) combination spheroids, whereas ES‐2 WT cells were unresponsive to HA. Previously we identified disabled‐2 (DAB2) as a novel target of 1000‐kDa HA and demonstrated Notch3 was required for upregulation of DAB2 in ES‐2 cells [6]. Further analysis of our mass spectrometry data identified sphingosine kinase 1 (SPHK1) to be regulated by both 183‐ and 1000‐kDa HA in our combination spheroids.

SPHK1 is involved in regulating the sphingolipid rheostat [8]. Sphingolipids are a class of bioactive lipids, which contain a sphingoid backbone [9, 10, 11]. They were initially thought to be purely structural; however, sphingolipids are now recognised as bioactive signalling molecules, in particular ceramide, sphingosine and sphingosine‐1‐phosphate (S1P) [9, 10, 11]. When cells are under stress, ceramide production is enhanced through either the de novo synthesis pathway or the salvage pathway and subsequently activates pro‐apoptotic pathways [9, 10, 11, 12]. Ceramide is catabolised by ceramidases to produce sphingosine. Sphingosine promotes apoptosis and regulates intra‐cellular calcium levels by promoting endosomal and lysosomal calcium release [13]. Sphingosine can be phosphorylated to S1P by SPHK1 or SPHK2. S1P can either interact with proteins within the cells or be transported across the plasma membrane to elicit pro‐tumourigenic signals including enhanced cell proliferation and survival, therapy resistance, invasion, motility and angiogenesis [14]. Hence, the balance of pro‐survival and pro‐apoptotic sphingolipids and their enzymes can contribute to cancer initiation, progression and chemoresistance.

HA activates multiple pro‐tumourigenic signalling pathways throughout cancer initiation, progression and therapy resistance. Due to the pro‐tumourigenic nature of both HA and SPHK1, we further examined the relationship between SPHK1 and ovarian cancer. Utilising online databases, we found enhanced expression of *SPHK1* in ovarian cancer compared to normal tissues. Furthermore, *SPHK1* expression is enhanced in metastatic versus primary ovarian cancer tissues, and high *SPHK1* is associated with poor outcome in HGSOC patients. In a tissue microarray (TMA) HGSOC cohort, epithelial SPHK1 was associated with reduced progression‐free survival (PFS). SPHK1 protein levels were increased in metastatic tissues and in matching relapse tissues compared to tissues at diagnosis. Our study identified a novel relationship between HA signalling and SPHK1 expression.

## 2 Materials and Methods

### 2.1 Cell Culture

CaOV3 and A2780 human ovarian cancer cell lines were purchased from European Collection of Authenticated Cell Cultures (ECACC, Salisbury, UK). ES‐2 human ovarian cancer cell line was provided by Dr. H. Albrecht (University of South Australia). All cell lines were verified by short tandem repeat (STR) testing in 2021 (Promega GenePrint10; Griffith University DNA sequencing facility, QLD, Australia). Cells were maintained in RPMI‐1640 (ES‐2 and A2780, 11875093, Life Technologies, USA) or DMEM (CaOV3, Life Technologies) media supplemented with 10% foetal bovine serum (FBS, Bovogen Biologicals, Australia) and antibiotics (100 U penicillin G, 100 μg/mL streptomycin sulphate and 0.25 μg/mL amphotericin B, Sigma‐Aldrich, USA) and maintained at 37°C in 5% CO_2_ environment.

### 2.2 Viral Transduction

Retrovirus containing pQCVIP vector (TAKARA, Japan), empty (Rv‐Ctrl) or encoding NICD3 (Rv‐NICD3), was prepared as described previously [6]. ES‐2 cells were transduced using Rv‐Ctrl or Rv‐NICD3 with polybrene (2 μg/mL, Sigma‐Aldrich) and stable clones were selected by incubating with puromycin (1 μg/mL, Sigma‐Aldrich) for 3 days.

### 2.3 Liquid Chromatography With Tandem Mass Spectrometry (LC–MS/MS)

ES‐2 cells were combined with ES‐2‐Rv‐NICD3 cells at a ratio of 1:3 and plated at 10,000 cells/well on 24‐well polyHEMA plates with vehicle control, 27‐, 183‐ or 1000‐kDa HA (50 μg/mL, Contipro Inc.). Spheroids were cultured for 72 h and isolated with a 40‐μm cell strainer (Pluriselect, Germany). Protein was extracted from spheroid samples and analysed by LC–MS/MS as described in a previous study (*n* = 2) [6].

### 2.4 Western Immunoblotting

ES‐2 WT, ES‐2:ES‐2‐Rv‐NICD3 (1:3), CaOV3 and A2780 ovarian cancer cells were plated at 2 × 10^5^ cells/well in 6‐well culture plates. Cells were cultured for 48 h before treatment with vehicle control (PBS) or 4‐methylumbelliferone (4‐MU, 1 mM, Sigma‐Aldrich) for 24 h. ES‐2 WT and ES‐2:ES‐2‐Rv‐NICD3 (1:3) spheroids (5 × 10^4^ cells/well) were cultured for 96 h before treatment for 24 h with 4‐MU or vehicle control. Lysates were prepared and protein (20 μg) electrophoresed as previously described [15]. Protein was detected with SPHK1 rabbit monoclonal antibody (1/1000, 12071S, Cell Signalling Technology, USA) and was detected as previously described [15]. GAPDH monoclonal mouse antibody (1/50,000, 60004‐1‐Ig, Proteintech) was used as a loading control.

### 2.5 Patient Tissue Cohort

TMA cohort was obtained with approval by the Royal Adelaide Hospital Human Ethics Committee (protocol number 140101 and 060903). TMAs were assembled from primary (*n* = 118) and metastatic (*n* = 49) HGSOC tissues for patients diagnosed between 1988 and 2013 (*n* = 133) (Table S1, matched primary and metastatic tissues: *n* = 36) [16]. Each patient tumour had duplicate or triplicate 1‐mm diameter cores. Paraffin‐embedded tissues, including normal ovaries (*n* = 9), benign serous cystadenomas (*n* = 6) and HGSOC tumours (*n* = 16) collected between 2008 and 2018, were also assessed (Table S2). Additional samples included matched HGSOC tissues at diagnosis and following relapse (*n* = 3, Table S3).

### 2.6 Immunohistochemistry

SPHK1 was detected by immunohistochemistry as previously described using citric buffer (pH 6) antigen retrieval [17]. Slides were incubated overnight at 4°C with SPHK1 rabbit polyclonal antibody (1:100, 10670‐1‐AP, Proteintech). SPHK1 immunoreactivity was detected as previously described [17]. Slides were scanned by NanoZoomer Digital Pathology System (Hamamatsu Photonics, SZK, Japan). Stromal and epithelial SPHK1 immunoreactivity was quantitated in QuPath software (version 0.2.3) with mean *H*‐score assessed for 3–15 areas per core or tissue section by three independent assessors [18].

### 2.7 Online Databases

Kaplan–Meier Plotter (kmplot.com) contains Affymetrix microarray expression data and patient survival outcomes [19]. Survival curves comparing *SPHK1* expression (#219257_s_at; auto‐select best cut‐off) in HGSOC patients (grades 2 + 3) were assessed for PFS (*n* = 1029), post progression survival (PPS, *n* = 698) and overall survival (OS, *n* = 1144).

GENT2 (gent2.appex.kr) database was used to analyse the relationship between *SPHK1* expression in normal ovarian surface epithelium (OSE), fallopian tube (FT) and different sub‐types of ovarian cancer [20]. GPL570 platform (HG‐U133) ovarian cancer tissue (*n* = 1626) microarray data was analysed. Individual samples were reviewed and assigned as FT, OSE, HGSOC, LGSOC, endometrial, mucinous or clear cell sub‐types.

GSE2109 dataset was accessed via the GEO2R platform (ncbi.nlm.nih.gov/geo/geo2r) to assess *SPHK1* expression (219257_s_at) in primary (*n* = 140), metastatic (*n* = 66), HGSOC primary (*n* = 68) and HGSOC metastatic (*n* = 36) ovarian tumours.

Co‐expression analysis of *SPHK1* expression in the TCGA firehose dataset including RNA sequencing (*n* = 307), microarray (*n* = 558) and U133 microarray (*n* = 535) platforms was assessed in cBioportal [21]. Spearman correlation coefficient (±0.4) and *q*‐value (0.05) cut‐offs were applied. Genes that met the thresholds and were consistent across all three platforms were assessed further (*n* = 268). The 268 genes were run through the DAVID functional annotation tool (david.ncifcrf.gov/) and the top 5 significant gene ontology (GO) term biological process (BP) direct (GOTERM_BP_DIRECT) hits were identified. The gene list was assessed by STRING mapper (string‐db.org/) and the top 5 significant GOTERM_BP_DIRECT hits were highlighted.

ROCPlotter (rocplot.org/ovarian) was accessed to analyse *SPHK1* (219257_s_at) expression in patients with grade 3 serous ovarian cancer who received combination platinum and taxane‐based chemotherapy treatment (*n* = 585). Expression between patients who experienced relapse (non‐responders) and did not experience relapse (responders) within 6 months of completing treatment was compared by Mann–Whitney test and ROC curve.

### 2.8 Patient‐Derived Explant Assay

HGSOC patient tissues were collected at surgery with approval from the Royal Adelaide Hospital Human Ethics Committee after informed consent (RAH protocol number 140101 and 060903, Table S4). Tissues were treated with 4‐MU (1 mM) or vehicle control (PBS) for 48 h and harvested and processed as described previously [17, 22]. SPHK1 expression in treated tissues was assessed by IHC.

### 2.9 Statistical Analysis

TMA Kaplan–Meier survival analyses were performed using IBM SPSS Statistics software (Version 28.0.1.0, IBM Corporation, USA). All other comparisons were performed in Prism for MacOS (Version 9.3.1, GraphPad, USA) including un‐paired Student's *t*‐test for data that was normally distributed. Non‐parametric statistical tests, including Wilcoxon rank paired test, Mann–Whitney and Kruskal–Wallis test followed by Dunn's multiple comparison, were used for data that was not normally distributed. Statistical significance was accepted at *p* < 0.05. (*p < 0.05; **p < 0.01; ***p < 0.001; ****p < 0.0001).

## 3 Results

### 3.1 Identification of SPHK1 as a Target of HMW‐HA Signalling

**FIGURE 1:**
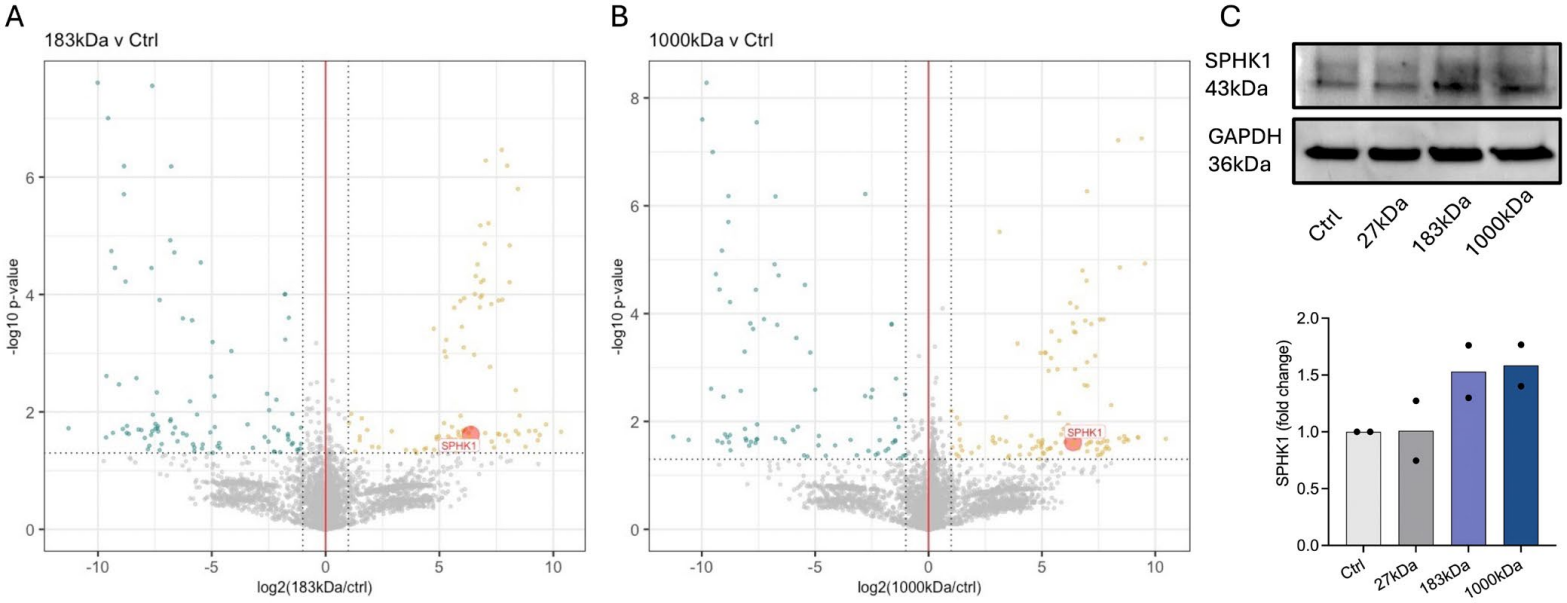
Mass spectrometry analysis of ES‐2:ES‐2‐Rv‐NICD3 (1:3) spheroids treated with control (ctrl), 183‐ or 1000‐kDa HA. Volcano plots depicting changes in protein expression in (A) 183‐kDa HA and (B) 1000‐kDa HA treated spheroids compared to ctrl treated spheroids. (C) Western blot analysis and quantitation of SPHK1 expression in ES‐2:ES‐2‐Rv‐NICD3 (1:3) spheroids treated with ctrl, 27‐, 183‐ and 1000‐kDa HA. Expression is normalised to house‐keeper GAPDH and presented as fold change compared to ctrl (*n* = 2 experiments).

In a recent study, we assessed the effects of different molecular weight HA in a population of ES‐2 cells over‐expressing NICD3 [6]. We identified DAB2 as a protein significantly enhanced by 1000‐kDa HA [6]. Furthermore, we demonstrated 1000‐kDa HA enhanced DAB2 expression in spheroids formed by OV90 and OVCAR3 HGSOC cells [6]. In the same study, we also identified SPHK1 as a protein regulated by 183‐ and 1000‐kDa HA signalling (Figure 1A,B). We confirmed 183‐ and 1000‐kDa HA enhanced expression of SPHK1 in ES‐2‐Rv‐NICD3 combination spheroids by immunoblotting (Figure 1C).

**FIGURE 2:**
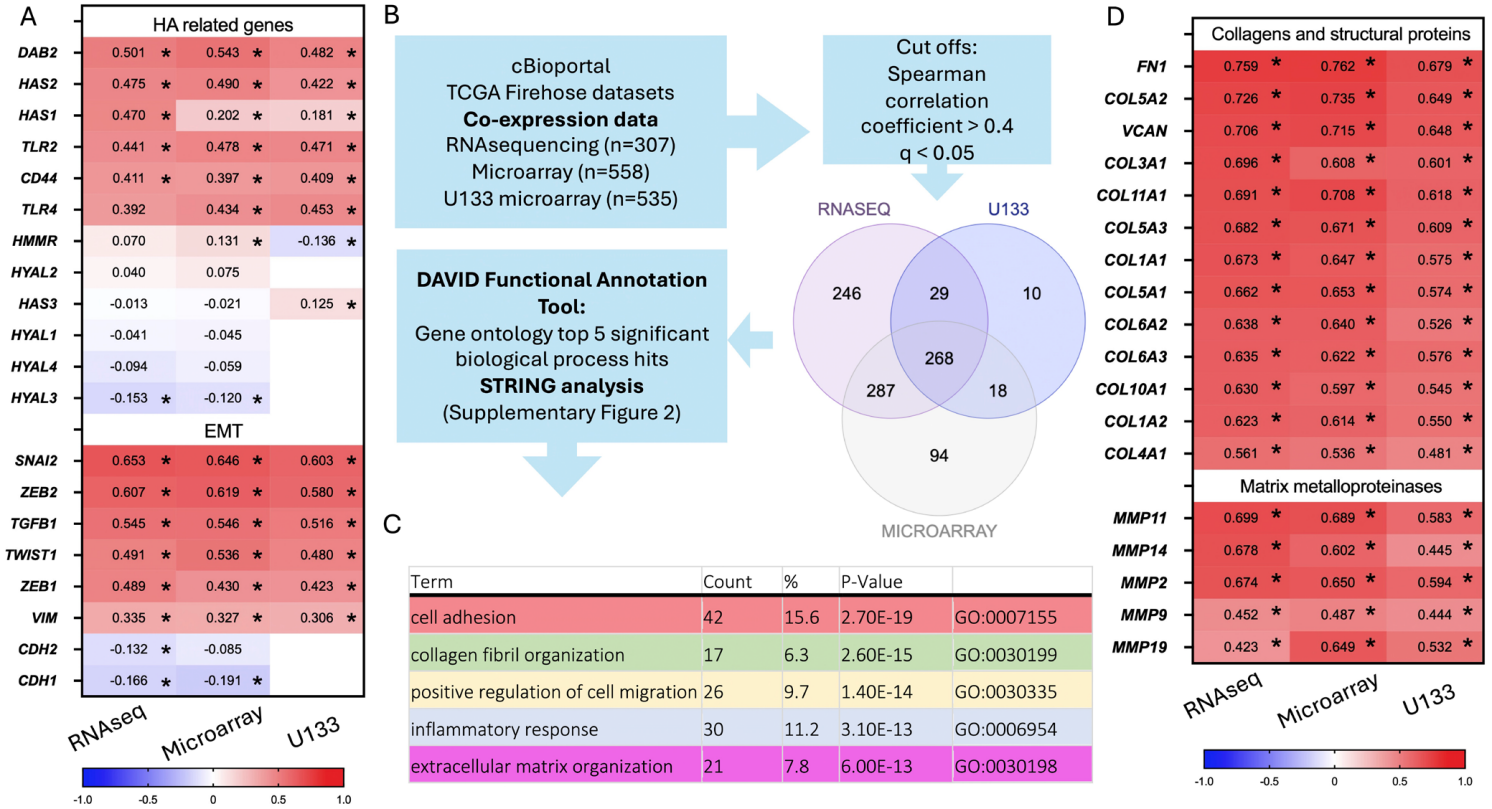
*SPHK1* expression correlates with genes involved in HA regulation, epithelial mesenchymal transition (EMT) and extracellular matrix (ECM) organisation in serous ovarian cancer. (A) Heat map of Spearman's rank correlation coefficients for *SPHK1* and HA and EMT related genes. Coefficients were isolated from cBioportal database co‐expression analysis of TCGA firehose dataset RNA sequencing (*n* = 307), microarray (*n* = 558) and U133 microarray (*n* = 535) gene expression platforms. Values = *R* coefficients, *p < 0.05. (B) Flow chart to assess the top biological processes related to *SPHK1* expression in ovarian cancer patient tumours. (C) The top 5 significant GOTERM_BP_DIRECT hits for genes with *R* ± 0.4 and *q* < 0.05 with *SPHK1* expression in both RNA sequencing and microarray gene expression platforms. (D) Heat map of Spearman's rank correlation coefficients for *SPHK1* and collagens, structural proteins and matrix metalloproteinases.

As HA is associated with tumour initiation and progression, we assessed the relationship between *SPHK1* and co‐expressed genes in ovarian cancer tissues (TCGA firehose dataset). There was a strong positive correlation between *SPHK1* and HA synthesis (*HAS1*, *HAS2*) and HA receptor (*CD44*, *TLR2*, *TLR4*) genes and *DAB2*, which we previously showed was regulated by HA (Figure 2A) [6]. We also found a positive relationship with *SPHK1* and genes associated with epithelial to mesenchymal transition (EMT) (Figure 2A). Significant positive correlations with mesenchymal markers and transcription factors (*SNAI2*, *ZEB2*, *TWIST1*, *TGFB1*, *ZEB1* and *VIM*) (Figure 2A) and a negative relationship with epithelial marker *CDH1* and mesenchymal *CDH2* were observed (Figure 2A). We assessed the top *SPHK1* co‐expressed genes in cBioportal (Figure 2B). There were 268 common genes across the three expression platforms that met thresholds for Spearman's rank correlation coefficient (0.4) and *q*‐value (0.05) (Figure 2B). The five most significant GOTERM_BP_DIRECT hits were cell adhesion, collagen fibril organisation, positive regulation of cell migration, inflammatory response and extracellular matrix (ECM) organisation (Figure 2C, Figure S1). There was a strong positive correlation with the ECM related proteins, including multiple collagen genes, matrix metalloproteinases (MMPs) and structural proteins (*FN1*, *VCAN*) (Figure 2D).

### 3.2 Inhibition of HA Synthesis by 4‐MU Inhibits SPHK1 Expression in Ovarian Cancer Cell Lines and HGSOC Patient Tissues

**FIGURE 3:**
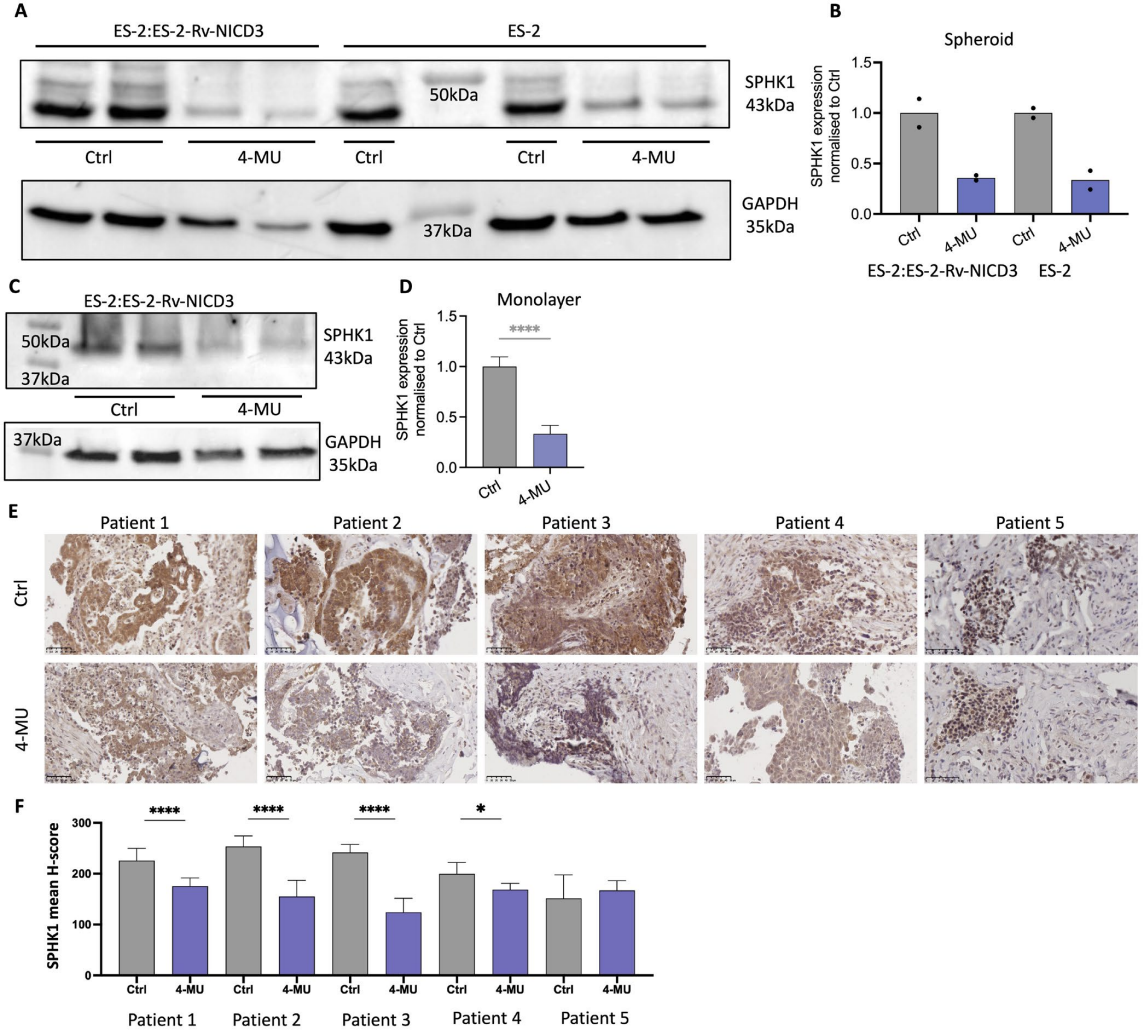
4‐methylumbelliferone (4MU) decreases SPHK1 expression in ovarian cancer cell lines and HGSOC patient tissues. (A) Western immunoblot analysis of SPHK1 protein levels in ES‐2:ES‐2‐Rv‐NICD3 (1:3) and ES‐2 WT spheroids treated with control (ctrl, PBS) or 4‐MU (1 mM) for 24 h (*n* = 2). (B) Quantitation of SPHK1 protein level in spheroids normalised to loading control GAPDH and ctrl (PBS) (*n* = 4, 2 experiments, mean ± SD). (C) Western immunoblot analysis of SPHK1 protein levels in ES‐2:ES‐2‐Rv‐NICD3 (1:3) monolayer cultures treated with ctrl (PBS) or 4‐MU (1 mM) for 24 h. (D) Quantitation of SPHK1 protein levels in ES‐2:ES‐2‐Rv‐NICD3 (1:3) monolayer cultures. (E) Representative images of SPHK1 expression in HGSOC patient‐derived explant assays treated with either ctrl (PBS) or 4‐MU (1 mM) for 48 h. Scale bar = 50 μm. (F) Quantitation of SPHK1 in HGSOC patient‐derived tissue explants using QuPath (*n* = 5, 5–15 sections per tissue, mean ± SD). ****p < 0.0001, ***p < 0.001, **p < 0.01, *p < 0.05; unpaired *t*‐test (D) and Mann–Whitney test (F).

To confirm the relationship between SPHK1 and HA, we assessed the effects of the HA synthesis inhibitor, 4‐MU on SPHK1 protein levels in ovarian cancer cells. 4‐MU treatment (1 mM) decreased SPHK1 in ES‐2:ES‐2‐Rv‐NICD3 (1:3) cells in spheroid (Figure 3A,B, 0.36‐fold change) and monolayer (Figure 3C,D, 0.33‐fold change, *p* < 0.0001) culture. To determine if NICD3 was required for HA‐induced effects on SPHK1, we assessed the effects of 4‐MU on ES‐2 WT cells. 4‐MU treatment decreased SPHK1 expression in ES‐2 WT spheroids (Figure 3A,B, 0.34‐fold change) and monolayer (Figure S2A, 0.3‐fold change, *p* = 0.0073) culture. 4‐MU also significantly decreased SPHK1 expression in CaOV3 (Figure S2B, 0.23‐fold change, *p* = 0.002) and A2780 ovarian cancer cells in monolayer culture (Figure S2C, 0.42‐fold, *p* = 0.00638). We additionally assessed the effects of 4‐MU on SPHK1 expression using a patient‐derived tissue explant assay (Figure 3E). 4‐MU significantly decreased SPHK1 expression in 4/5 of patient tissues (Figure 3E,F). No effects of 4‐MU treatment were observed in patient 5, who had the lowest SPHK1 expression (Figure 3E,F).

### 3.3 SPHK1 Expression Is Increased in Metastatic Ovarian Cancer and Following Relapse in HGSOC Tissues

**FIGURE 4:**
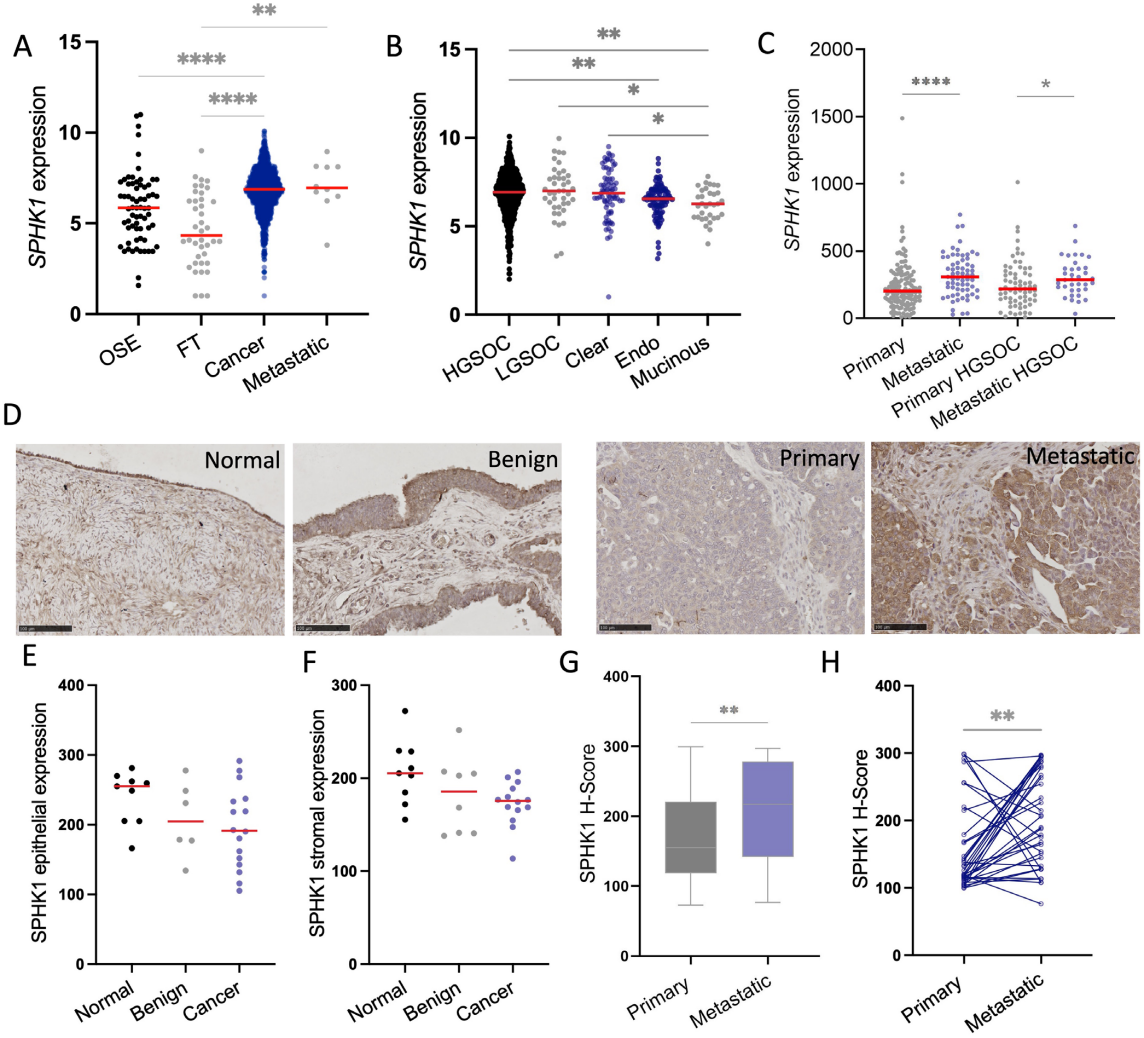
SPHK1 expression is increased in metastatic ovarian cancer compared to primary ovarian cancer. GENT2 online gene expression database analysis of *SPHK1* expression in (A) OSE (*n* = 66), FT (*n* = 40), cancer (*n* = 1119) and metastatic tissues (*n* = 10) and (B) HGSOC (*n* = 803), LGSOC (*n* = 41), clear cell (*n* = 77), endometrioid (endo, *n* = 101) and mucinous (*n* = 33) ovarian tumours. (C) *SPHK1* expression from online dataset GSE2109 in primary (*n* = 140) and metastatic (*n* = 66) ovarian cancer tissue and primary HGSOC (*n* = 68) and metastatic HGSOC (*n* = 36) tissues. (D) Representative images of SPHK1 immunostaining (1/100, Proteintech, 10670‐1‐AP) in normal ovary, benign ovarian cancer, primary HGSOC and matched metastatic HGSOC. Scale bar = 100 μm. (E) Epithelial and (F) stromal SPHK1 immunoreactivity *H*‐score in normal (*n* = 9), benign (*n* = 8) and HGSOC tissues (*n* = 16). (G) SPHK1 *H*‐score in primary (*n* = 115) and metastatic (*n* = 49) HGSOC tissues from the TMA cohort. (H) SPHK1 *H*‐score in matched primary and metastatic HGSOC tissues (*n* = 36). ****p < 0.0001, ***p < 0.001, **p < 0.01, *p < 0.05; Kruskal–Wallis test (A, B) and Mann–Whitney test (C, G), Wilcoxon test (H).

We analysed *SPHK1* gene expression in OSE, FT, different ovarian cancer types and metastatic ovarian cancer tissues in the GENT2 database. *SPHK1* expression was significantly increased in ovarian cancer compared to OSE and FT (Figure 4A, ****p < 0.0001) and in metastatic tissues compared to FT (Figure 4A, **p = 0.0013). *SPHK1* expression was significantly increased in HGSOC and LGSOC compared to other sub‐types (Figure 4B). *SPHK1* expression was significantly increased in metastatic compared to primary ovarian cancer tissues (Figure 4C, ****p < 0.0001) and in metastatic HGSOC compared with primary HGSOC tissues (Figure 4C, *p = 0.0301). We also assessed SPHK1 protein abundance in normal ovary, benign serous cystadenoma, HGSOC and a TMA cohort of primary and metastatic HGSOC by immunohistochemistry (Figure 4D). Quantitation of SPHK1 immunostaining showed no significant differences in SPHK1 epithelial (Figure 4E) and stromal (Figure 4F) expression in normal ovary, benign ovarian cancer and HGSOC tissues. Epithelial SPHK1 abundance was significantly increased in metastatic tissues compared to primary ovarian carcinoma (Figure 4G, *p* = 0.0033) and in metastatic HGSOC tissues compared to matched primary HGSOC tissues (Figure 4H, *p* = 0.0049).

**FIGURE 5:**
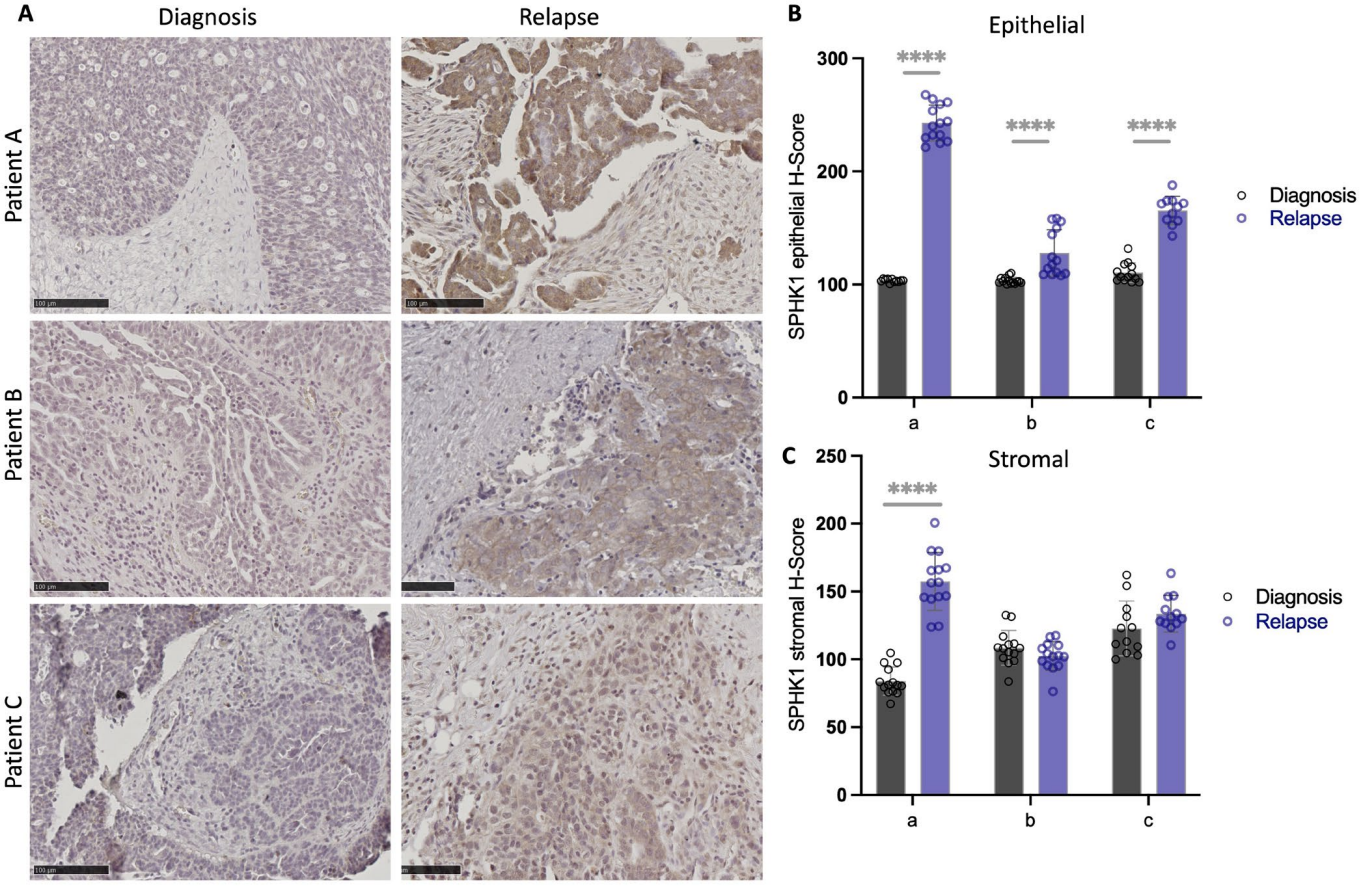
Epithelial SPHK1 expression is enhanced in HGSOC at relapse compared to matching HGSOC at diagnosis. (A) Representative images of SPHK1 immunostaining in matched HGSOC patient tissues at relapse and diagnosis. Scale bar = 100 μM. Quantitation of SPHK1 expression in (B) epithelial and (C) stroma in matched HGSOC patient tissues at diagnosis and relapse. (****p < 0.0001, unpaired *t*‐test, *n* = 10–16 areas assessed per tissue).

We assessed SPHK1 protein levels in matched HGSOC tissues at relapse and diagnosis (Figure 5A). SPHK1 epithelial abundance was significantly increased in relapse tissues compared to matched diagnosis tissues (Figure 5B, patient A: 2.35‐fold, patient B: 1.23‐fold and patient C: 1.5‐fold; ****p < 0.0001). Stromal SPHK1 levels were significantly increased in relapse tissues compared to diagnosis in patient A (Figure 5C, 1.88‐fold, ****p < 0.0001) but not for patients B and C.

### 3.4 SPHK1 Expression Is Associated With Poor Prognosis in Patients With HGSOC

**FIGURE 6:**
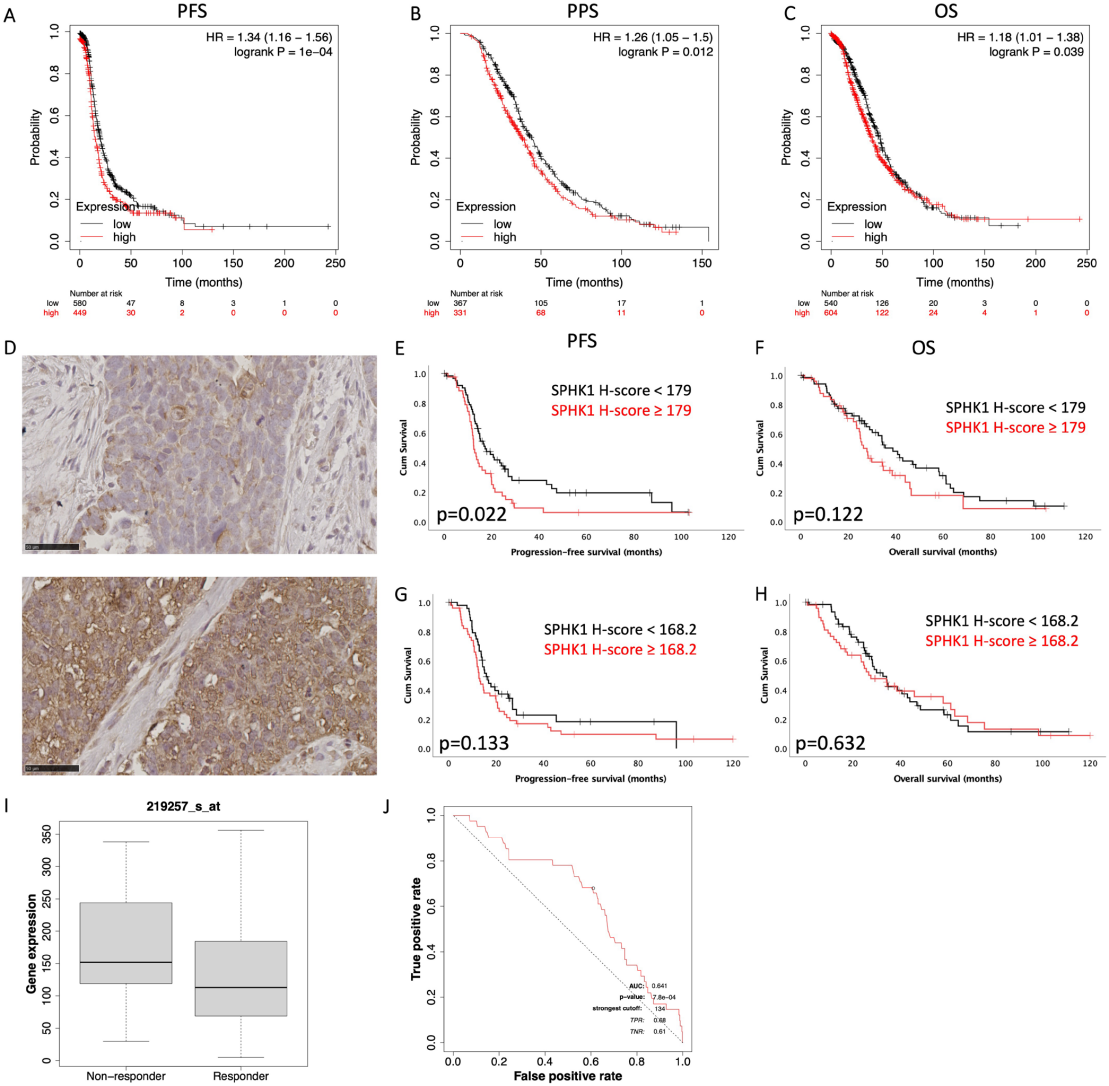
High SPHK1 levels are associated with poor prognosis and therapy resistance in HGSOC patients. Kaplan–Meier survival plots for *SPHK1* expression and (A) PFS (*n* = 1029), (B) PPS (*n* = 698) and (C) OS (*n* = 1144) in HGSOC patients. (D) Representative images of low (top) and high (bottom) SPHK1 immunostaining in the TMA cohort. Kaplan–Meier survival plots for epithelial SPHK1 protein level (mean *H*‐score cut off 179) and (E) PFS and (F) OS in HGSOC patients. Kaplan–Meier survival plots for SPHK1 protein level in tumour‐associated stroma (mean *H*‐score cut off 168.2) and (G) PFS and (H) OS in HGSOC patients. ROCplotter analysis of (I) *SPHK1* expression in serous ovarian cancer patients (grade 3) who received combination platinum and taxane‐based chemotherapy and experienced relapse (non‐responders) and patients who didn't relapse (responders) within 6 months of completing treatment. (J) ROC analysis of *SPHK1* expression as an indicator of platinum and taxane chemotherapy sensitivity in HGSOC patients.

Analysis of Kaplan–Meier Plotter (kmplot.com) microarray expression found high *SPHK1* expression was associated with significantly reduced PFS (Figure 6A, HR: 1.34 [1.16–1.56], *p* = 1e‐04), PPS (Figure 6B, HR: 1.26 [1.05–1.5], *p* = 0.012) and OS (Figure 6C, HR: 1.18 [1.01–1.38], *p* = 0.039) in HGSOC. To verify the relationship between high *SPHK1* expression and poor prognosis in HGSOC patients, we additionally assessed SPHK1 protein levels in a HGSOC TMA cohort. Representative images of low and high SPHK1 immunostaining are shown in Figure 6D. Patients with epithelial SPHK1 staining greater than the mean *H*‐score (179) had significantly reduced PFS (Figure 6E, *p* = 0.022). There was no significant relationship between epithelial SPHK1 and OS (Figure 6F, *p* = 0.122). We observed no significant relationship between SPHK1 protein levels in the tumour‐associated stroma with either PFS or OS (Figure 6G,H). We analysed the online database ROCplotter to determine if *SPHK1* expression was associated with therapy resistance. We compared *SPHK1* gene expression in HGSOC patients (grade 3) in responders and non‐responders within 6 months of completing platinum and taxane chemotherapy treatment. *SPHK1* expression was significantly increased in HGSOC tumours from patients who experienced relapse (non‐responders) compared to those who did not relapse (responders) (Figure 6I, *p* = 0.0026). The ROC curve analysis confirmed a significant relationship between SPHK1 expression and therapy response (Figure 6J, AUC = 0.641, *p* = 0.00078).

## 4 Discussion

HA is an important structural and signalling element within the ECM, involved in initiation, progression and relapse of tumours [3]. Although the effects of HA are well studied in cancer, the role of different molecular weight HA is not well defined. In a recent study, we assessed the effects of different molecular weight HA in a population of ES‐2 ovarian cancer cells over‐expressing NICD3 to simulate a stem‐like population [6]. We showed a protein, DAB2, was significantly upregulated by 1000‐kDa HA in a Notch3 dependent manner. Further analysis of our data found SPHK1 protein expression was increased following treatment with HMW‐HA (183 and 1000 kDa). In this study, we confirmed that SPHK1 protein levels were increased by 183‐ and 1000‐kDa HA in ES‐2 spheroids over‐expressing NICD3. Furthermore, the HA synthesis inhibitor 4‐MU reduced SPHK1 protein levels in ovarian cancer cells and HGSOC patient tissues using ex vivo tissue explant assays. Previous biophysical assays have demonstrated that HA polymers of increasing molecular weight have enhanced binding affinity for receptor CD44, and furthermore promote enhanced clustering of CD44 at the plasma membrane [23]. We observed positive correlations between *SPHK1* expression and CD44 in ovarian cancer patient tissues. Additionally, SPHK1 was positively correlated with HA synthesis enzymes (*HAS1* and *HAS2*) involved in producing HMW‐HA polymers [24]. Together, these findings support the relationship between HMW‐HA and SPHK1 expression.

To the best of our knowledge, this is the first study to demonstrate a relationship between HMW‐HA and SPHK1 expression; however, other studies have found relationships between HA and other components of the sphingolipid pathway. SPHK1 phosphorylates sphingosine to form S1P, a bioactive signalling molecule that has been implicated in promoting pro‐tumourigenic signals including angiogenesis, cell survival, motility and invasion [25]. Different molecular weight HA polymers have been shown to differentially regulate the sphingolipid rheostat between pro‐tumourigenic S1P signalling and tumour suppressive ceramide [26, 27, 28]. This study is the first to demonstrate a relationship between HMW‐HA signalling and the sphingolipid pathway in cancer. In endothelial cells, HMW‐HA (1000 kDa) promoted the interaction of CD44 with S1P1 receptor at the membrane, allowing transactivation of AKT signalling and increased barrier integrity [26]. Low molecular weight HA (LMW‐HA) 2.5 kDa has been shown to interact with a CD44 variant (CD44v10) and promote the interaction with barrier‐disrupting S1P3 receptor [26, 29]. These findings suggest that different CD44 isoforms can manipulate the sphingolipid rheostat through interactions with different molecular weight HA. In keratinocytes, HA tetra‐saccharides (LMW‐HA) were found to push the sphingolipid rheostat towards ceramide production [27]. In fibroblasts lacking neutral sphingomyelinase, decreased ceramides upregulated *HAS2* gene expression, activity and secretion of HA. Furthermore, in wild‐type fibroblasts, exogenous HA, 950 kDa, was found to protect against starvation‐induced apoptosis via expression of anti‐apoptotic heat shock protein 72 (Hsp72) [28, 30]. The correlation data from cBioportal online databases support a relationship between HA signalling and SPHK1. We observed significant positive correlations between the expression of hyaluronan synthases (*HAS1‐2*) and *SPHK1* in ovarian cancer patient tissues (TCGA RNAseq and microarray). HAS2 (> 2 × 10^6^ Da) and HAS1 (2 × 10^5^–1 × 10^6^ Da) produce HA polymers of higher molecular weight compared to HAS3 (1 × 10^5^–10^6^ Da), consistent with the observed effects by HMW‐HA [3]. Supporting the relationship between SPHK1 expression and exogenous HA, there were positive correlations between *SPHK1* and receptors *CD44*, *TLR2* and *TLR4*, which are located in the plasma membrane, whereas the receptor for hyaluronan mediated motility (RHAMM/*HMMR*), located in the cytoplasm and interacting with endogenous HA [3], had no significant correlation with *SPHK1*. Further studies utilising neutralising CD44 antibody and HA oligomers, which inhibit HA/CD44 interactions, will validate the involvement of CD44 in the HA/SPHK1 axis. We have previously demonstrated that the use of HA oligomers can inhibit the effects of exogenous HA on the viability of ovarian cancer cell lines [4]. A small 12mer peptide (GAHWQFNALTVR), PEP‐1, has been shown to inhibit the interactions of exogenous HA with TLR4 and CD44 [31]. A 15mer peptide (Pep‐15) which mimics the HA binding domain in RHAMM and competitively interacts with HA is also available to help tease out any involvement of RHAMM [32].

4‐MU treatment significantly reduced expression of SPHK1 in ES‐2, A2780 and OVCAR3 cells. 4‐MU has been shown to inhibit HA synthesis by sequestering substrate glucuronic acid and reducing expression of HA synthases (HAS2 and HAS3) [33]. However, 4‐MU is multi‐functional, with effects observed in cells with no HAS expression [33]. Recently, a novel HA synthase inhibitor has been designed, 5′‐deoxy‐5′‐(1,3‐diphenyl‐2‐imidazolidinyl)‐thymidine (DDIT) which has enhanced potency over 4‐MU, although its specificity is yet to be characterised [34]. To fully characterise the HA/SPHK1 axis in ovarian cancer, knockdown assays individually targeting HAS1‐3 are required. The use of hyaluronidase in future assays can also be applied to break down HA polymers in the pericellular coat to further verify the importance of exogenous HA in regulating SPHK1 expression. These additional experiments will further elucidate the role of HA in SPHK1 signalling.

Over‐expression of SPHK1 protein and gene expression compared to normal tissues has been observed in cancers of the bladder [35], oesophagus [36], colon [37], stomach [38], breast [39] and ovary [40]. GENT2 database showed significantly increased *SPHK1* expression in epithelial ovarian cancer tissue compared to OSE and FT. Another study utilising RT‐PCR for snap frozen ovarian cancer tissues found significantly elevated *SPHK1* gene expression in epithelial ovarian cancer compared to benign tissues [40]. We observed no significant difference in both epithelial and stromal SPHK1 protein abundance between OSE, benign ovarian tumours, and HGSOC tissues. Our assessment was only in a small cohort of HGSOC, which may account for the lack of significant differences observed. Furthermore, we only assessed SPHK1 protein in normal ovary and not FT, which is hypothesised as one of the primary sites for HGSOC initiation [41]. Different molecular sub‐types could also contribute to dissimilar findings. HGSOC, the most common histological sub‐type of ovarian cancer, can be further characterised [42, 43]. Large‐scale sequencing studies utilising clustering algorithms have identified four distinct molecular sub‐types including (C1) mesenchymal phenotype, (C2) immunoreactive phenotype with high immune infiltration, (C4) differentiated and (C5) proliferative [40, 42, 43]. *SPHK1* expression was previously found to be increased in the mesenchymal (C1) that has the poorest prognosis compared to the differentiated (C4) sub‐type that is associated with a better outcome [40].

Our DAVID analysis of the top *SPHK1* co‐expressed genes in ovarian cancer tissues (TCGA Firehose dataset, cBioportal) found positive relationships with cell adhesion, ECM organisation and positive regulation of cell migration. Consistent with this analysis, we observed enhanced SPHK1 gene and protein expression in metastatic compared to primary ovarian cancer tissues. Our previous work demonstrated that mislocalised SPHK1, caused by loss of a negative regulator protein, calcium and integrin binding protein 2 (CIB2), promoted tumour growth, metastasis and chemotherapeutic resistance in ovarian cancer cell lines and a xenograft mouse model [25]. Consistent with our findings, another study also found significantly elevated SPHK1 protein in omental metastases compared to matched primary HGSOC tumours [44]. Culture media from omental adipocytes significantly enhanced cell proliferation, migration and invasion of SKOV‐3 ovarian cancer cells via SPHK1 [8, 44]. In oesophageal cancer, high SPHK1 protein was associated with increased risk of lymph node metastasis, and SPHK1 promoted cell migration, invasion and in vivo tumourigenesis and lung metastases [36]. Similarly, in gastric cancer, high SPHK1 was associated with metastasis and venous invasion [38]. A breast cancer study also found a significant correlation between phosphorylated‐SPHK1 and lymph node metastases [39]. In HGSOC, relapse tumours frequently develop chemotherapy resistance [2]. SPHK1 promotes chemotherapy resistance in leukaemia [45], prostate [46] and breast cancer [47]. We observed significantly increased SPHK1 expression in relapse HGSOC compared to matched tissues at diagnosis. Additionally, *SPHK1* expression was increased in ovarian cancer patients (serous, grade III) who did not respond to combination platinum and taxane chemotherapy treatment. Considering the relationship between therapy resistance and SPHK1 in other cancers, we hypothesise that the elevated SPHK1 in relapse ovarian cancer may be contributing to therapy resistance in HGSOC.

A relationship between high SPHK1 and poor patient outcomes has been observed in bladder [35], breast [39], gastric [38], glioblastoma [48] and oesophageal [36] cancers. Our analysis of KMplot.com shows high *SPHK1* expression was significantly associated with reduced PFS, PPS and OS in HGSOC. Beach et al. and Teng et al. also showed using KMplot.com, high *SPHK1* was associated with reduced PFS and OS in serous ovarian cancer patients (all grades) [40, 49]. Considering KMplot microarray data encapsulates all areas of the tumour, including tumour‐associated stroma and tumour epithelium, we assessed SPHK1 protein levels further in a TMA cohort. High epithelial SPHK1 abundance was associated with significantly reduced PFS. We found no significant relationship for stromal SPHK1 expression and HGSOC prognosis. Surprisingly, another study with a large patient cohort (*n* = 613) found low SPHK1 protein expression was associated with significantly reduced PFS and OS in HGSOC patients [50]. The disparate findings may be due to differences in antibody specificity, as functional ovarian cancer studies support a relationship with SPHK1 and tumour progression [51, 52]. Hanker et al. [50] used a rabbit polyclonal antibody from IMGENEX (IMG‐72025), whereas our rabbit polyclonal antibody was purchased from Proteintech (10670‐1‐AP). Additionally, we used computational QuPath software to detect staining intensity whilst they manually scored the staining intensity. Further validation of SPHK1 protein abundance with different antibodies in larger tissue cohorts is required.

Targeting HA signalling has many difficulties; hyaluronidase treatments such as PEGPH‐20 have been assessed in phase III clinical trials to improve the efficacy of chemotherapeutics; however, limited benefits of PEGPH‐20 were observed [53]. 4‐MU is approved in Europe for the treatment of biliary spasms; however, its efficacy in cancer is yet to be assessed by clinical trials [54]. Due to the complexities of HA synthesis and the functions of different molecular weight HA, SPHK1 offers a more promising therapeutic target. Selective SPHK1 inhibitor (PF‐543) significantly reduced tumour formation and ascites volume in an ovarian cancer model using ID8 mouse OSE cells [51]. SPHK1 inhibition by PF‐543 or shRNA knockdown in SKOV3 cells also significantly reduced tumour formation and tumour metastases in a mouse xenograft [44]. A recent study showed SPHK1 inhibition by PF‐543 enhanced the sensitivity of ovarian cancer cells to Olaparib and reduced colony formation [49]. SPHK1 suppression has also been shown to reduce tumour burden in vivo for head and neck [52], colon [55], breast [56] and intestinal [57] cancers. Currently, there are over 30 inhibitors which target SPHK1 [58, 59]. FTY720 is FDA approved for the treatment of multiple sclerosis; however, no clinical trials have assessed its efficacy in cancer [58, 59]. Safingol, a dual SPHK1 and SPHK2 inhibitor, is the only SPHK1 inhibitor to reach clinical trial [58, 59]. The Phase I trial demonstrated safingol in combination with cisplatin for multiple solid tumour types was tolerated with manageable side effects [58, 59].

Both HA and SPHK1 are established in their involvement in tumour progression; however, this study is the first to demonstrate a relationship between HMW‐HA signalling and the sphingolipid pathway in cancer. We demonstrated that HMW‐HA increased SPHK1 expression and showed that inhibition of HA synthesis can reduce SPHK1 expression in cell lines and HGSOC patient tissues, but the mechanisms and pathways involved need further investigation. There is growing evidence that SPHK1 is a promising therapeutic target for multiple cancers, and this study offers a promising strategy for targeting SPHK1 in ovarian cancer patients.

## Author Contributions

**Zoe K. Price:** conceptualization (equal), data curation (lead), formal analysis (equal), funding acquisition (supporting), writing – original draft (lead). **Noor A. Lokman:** conceptualization (equal), data curation (equal), formal analysis (equal), writing – original draft (equal). **Jessica Morrison:** conceptualization (supporting), data curation (equal), formal analysis (equal), writing – original draft (equal). **Sisanda N. Mhlanga:** conceptualization (supporting), data curation (equal), formal analysis (equal), writing – original draft (equal). **Mai Sugiyama:** conceptualization (equal), data curation (equal), writing – original draft (equal). **Yoshihiro Koya:** conceptualization (supporting), writing – original draft (equal). **Lorena T. Davies:** conceptualization (supporting), writing – original draft (equal). **Stuart M. Pitson:** conceptualization (supporting), writing – original draft (equal). **Martin K. Oehler:** conceptualization (supporting), funding acquisition (equal), writing – original draft (equal). **Melissa R. Pitman:** conceptualization (supporting), writing – original draft (equal). **Masato Yoshihara:** conceptualization (equal), funding acquisition (equal), writing – original draft (equal). **Hiroaki Kajiyama:** conceptualization (equal), funding acquisition (equal), writing – original draft (equal). **Carmela Ricciardelli:** conceptualization (equal), data curation (equal), formal analysis (equal), funding acquisition (equal), writing – original draft (equal).

## Ethics Statement

Patient samples were collected with approval by the Royal Adelaide Hospital Human Ethics Committee (RAH protocol number 140101, 060903 and R20181215) and patient informed consent.

## Conflicts of Interest

The authors declare no conflicts of interest.

## Supporting information


**Figure S1.** STRING analysis of genes with strongest Spearman correlations with SPHK1 expression (*r* > 0.4, *q* < 0.05) in ovarian cancer patient tissues.
**Figure S2.** 4‐MU decreases SPHK1 expression in ovarian cancer cell lines.
**Table S1.** Clinicopathological characteristics of serous ovarian cancer TMA cohort.
**Table S2.** Clinicopathological characteristics of HGSOC patient tissues used for the explant assay.
**Table S3.** Clinicopathological characteristics of HGSOC‐matched patient tissues at diagnosis and relapse.

## Data Availability

The datasets used and/or analysed during the current study are available from the corresponding author on reasonable request.
