# Supplementary material for: Identification of Sphingosine Kinase 1 as a Novel Protein Regulated by High Molecular Weight Hyaluronan in Ovarian Cancer
Source: J Cell Mol Med. 2025 May 12;29(9):e70574. doi: 10.1111/jcmm.70574 (PMC12069020; doi:10.1111/jcmm.70574)

**Supplementary Table S1.** Clinicopathological characteristics of serous ovarian cancer TMA cohort

| **Primary High grade serous ovarian carcinomas (n=118)** | | |
| --- | --- | --- |
| Age at Diagnosis (years) | Median (range) | 60 (25-87) |
| Histological Grade | Grade 2  Grade 3 | 20  98 |
| FIGO stage | Stage II  Stage III  Stage IV | 2  110  6 |
| SPHK1 H-score Ep | Median (range)  Mean ± SD | 157.4(72.8-299.5) 178.6 ± 65.1 |
| SPHK1 H-score St | Median (range)  Mean ± SD | 168.2(60.2-296.8) 155.5 ± 60.9 |
| Recurrence | No  Yes  Unknown | 23  85  10 |
| Cause of Death | Ovarian cancer  Other cause  Alive  Lost to follow-up | 74  10  33  1 |
| **Metastatic High grade serous ovarian carcinomas (n=49)** | | |
| Age at Diagnosis (years) | Median (range) | 69 (45-90) |
| Histological Grade | Grade 2  Grade 3 | 7  42 |
| FIGO stage | Stage II  Stage III  Stage IV | 0  44  5 |
| SPHK1 H-score Ep | Median (range)  Mean ± SD | 217(76.5-297)  212.4 ± 70 |
| Recurrence | No  Yes  Unknown | 14  30  5 |
| Cause of Death | Ovarian cancer  Other cause  Alive  Lost to follow-up | 25  3  12  8 |

**Supplementary Table S2:** Clinicopathological characteristics of normal, benign serous cystadenoma and HGSOC patient tissues used for the explant assay.

| **Normal (n=9)** | | |
| --- | --- | --- |
| Age | Median (range) | 46 (39-62) |
| **Benign serous cystadenoma (n=6)** | | |
| Age at diagnosis | Median (range) | 62 (25-80) |
| **HGSOC (n=16)** | | |
| Age at Diagnosis | Median (range) | 60 (42-81) |
| Histological grade | Grade 3 | 16 |
| FIGO Stage | Stage I  Stage II  Stage III | 1  1  14 |
| Recurrence | No  Yes | 10  6 |
| Cause of Death | Ovarian Cancer  Other cause  Alive | 5  3  9 |

**Supplementary Table S3:** Clinicopathological characteristics of HGSOC matched patient tissues at diagnosis and relapse

| Matched diagnosis and relapse serous carcinomas (n=3) | | |
| --- | --- | --- |
| Age at Diagnosis | Median (range) | 46 (42-62) |
| Age at relapse | Median (range) | 51(46-65) |
| FIGO stage | Stage I | 1 |
|  | Stage III | 2 |
| Histological Grade | Grade 3 | 3 |
| Cause of Death | Ovarian cancer | 1 |
|  | Alive | 1 |
|  | No follow-up | 1 |

**Supplementary Table S4:** Clinicopathological characteristics of HGSOC patient tissues used for the explant assay

| Patient | Age at diagnosis (years) | Stage at Diagnosis | Grade | Diagnosis |
| --- | --- | --- | --- | --- |
| 1 | 47 | IIIC | 3 | Serous carcinoma of the peritoneum |
| 2 | 63 | IIIC | 3 | Serous carcinoma of the peritoneum |
| 3 | 66 | IIIC | 3 | Serous papillary carcinoma of the ovary |
| 4 | 80 | IIIC | 3 | Primary peritoneal carcinoma |
| 5 | 80 | IIIC | 3 | Serous papillary carcinoma of the ovary |

**Supplementary Fig. S1:** STRING analysis of genes with strongest spearman correlations with *SPHK1* expression (r>0.4, q<0.05) in ovarian cancer patient tissues (TCGA Firehose, cBioportal). Top GO gene ontology terms were cell adhesion (red), collagen fibril organization (green), positive regulation of cell migration (yellow), inflammatory response (blue) and extracellular matrix organization (pink).


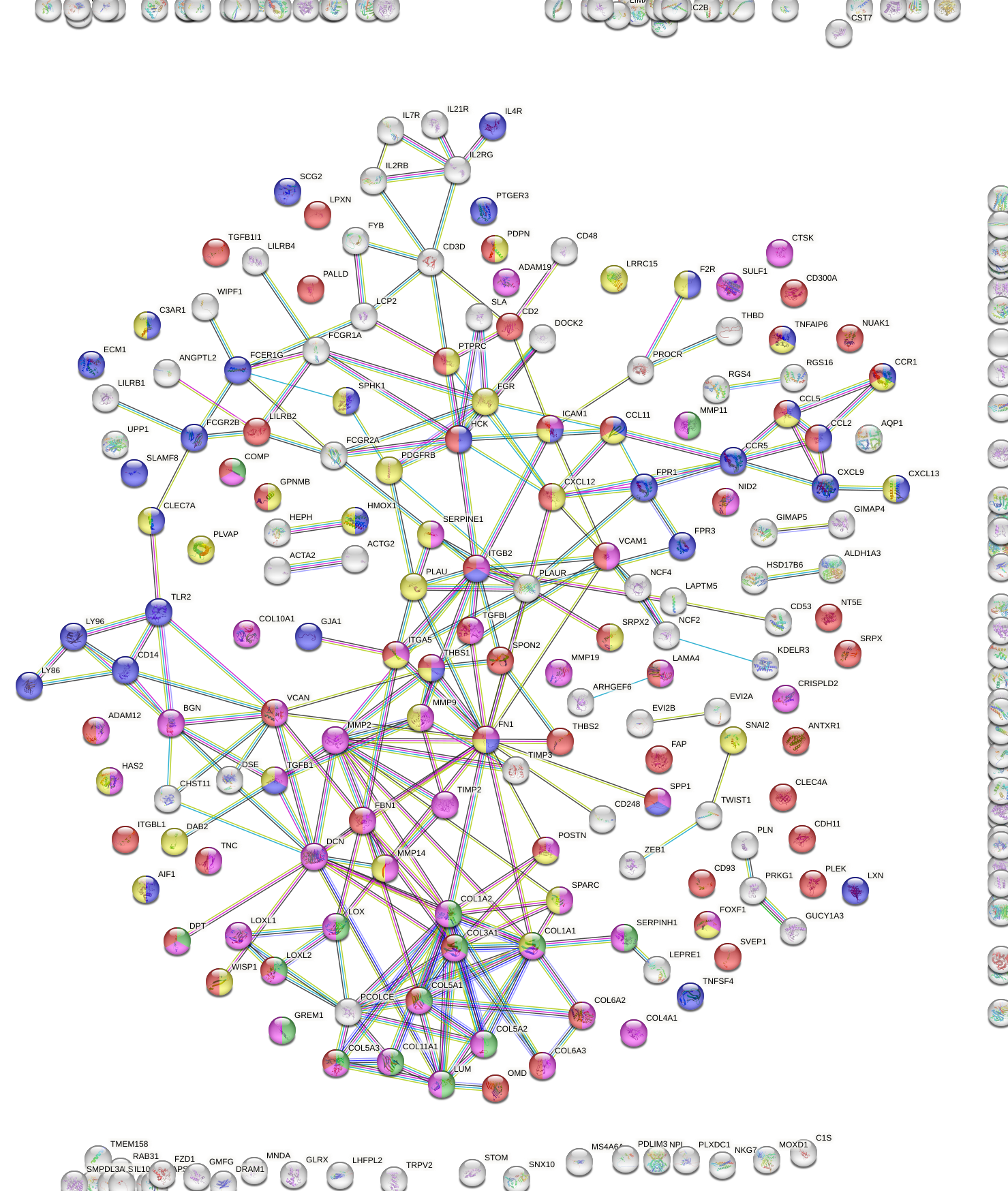


**Supplementary Fig.S2.** 4-MU decreases SPHK1 expression in ovarian cancer cell lines. Western immunoblot of SPHK1 expression in **A** ES-2, **B** CaOV3 and **C** A2780 monolayer cultures treated with 4-MU (1mM) for 24hr (3 experiments in duplicate). **p<0.01, ***p<0.001, Statistical analysis un-paired t-test.


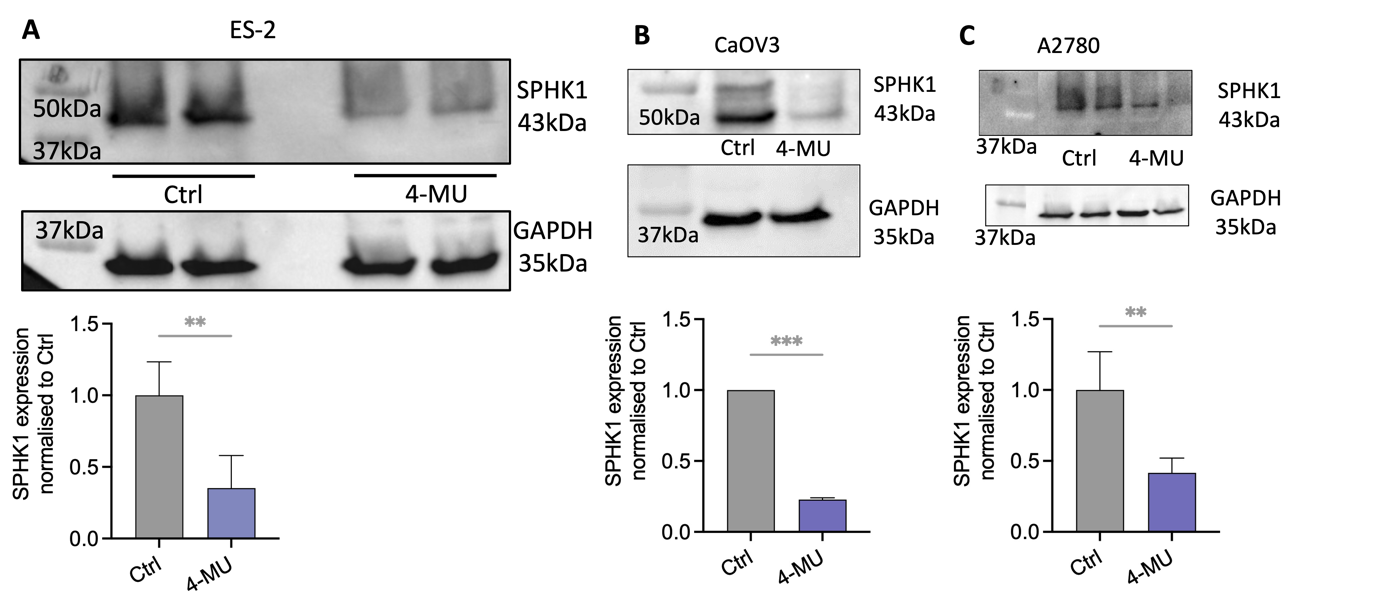

Supplement: Supplementary file 1 — Figure S1. STRING analysis of genes with strongest Spearman correlations with SPHK1 expression (r > 0.4, q < 0.05) in ovarian cancer patient tissues. Figure S2. 4‐MU decreases SPHK1 expression in ovarian cancer cell lines. Table S1. Clinicopathological characteristics of serous ovarian cancer TMA cohort. Table S2. Clinicopathological characteristics of HGSOC patient tissues used for the explant assay. Table S3. Clinicopathological characteristics of HGSOC‐matched patient tissues at diagnosis and relapse. [file JCMM-29-e70574-s001.docx]
